# Grow fast at no cost: no evidence for a mortality cost for fast early-life growth in a hunted wild boar population

**DOI:** 10.1007/s00442-020-04633-9

**Published:** 2020-04-02

**Authors:** Lara Veylit, Bernt-Erik Sæther, Jean-Michel Gaillard, Eric Baubet, Marlène Gamelon

**Affiliations:** 1 Centre for Biodiversity Dynamics, Department of Biology, Norwegian University of Science and Technology, 7491 Trondheim, Norway; 2 Laboratoire de Biométrie et Biologie Évolutive (UMR 5558), Université Claude Bernard Lyon 1, 43 boulevard du 11 novembre 1918, 69622 Villeurbanne Cedex, France; 3 Office Français de la Biodiversité, Unité Ongulés Sauvages, Montfort, 01330 Birieux, France

**Keywords:** Capture–mark–recapture analysis, Covariation in life-history traits, Early-life growth, Exploited population

## Abstract

From current theories on life-history evolution, fast early-life growth to reach early reproduction in heavily hunted populations should be favored despite the possible occurrence of mortality costs later on. However, fast growth may also be associated with better individual quality and thereby lower mortality, obscuring a clear trade-off between early-life growth and survival. Moreover, fast early-life growth can be associated with sex-specific mortality costs related to resource acquisition and allocation throughout an individual’s lifetime. In this study, we explore how individual growth early in life affects age-specific mortality of both sexes in a heavily hunted population. Using longitudinal data from an intensively hunted population of wild boar (*Sus scrofa*), and capture–mark–recapture–recovery models, we first estimated age-specific overall mortality and expressed it as a function of early-life growth rate. Overall mortality models showed that faster-growing males experienced lower mortality at all ages. Female overall mortality was not strongly related to early-life growth rate. We then split overall mortality into its two components (i.e., non-hunting mortality vs. hunting mortality) to explore the relationship between growth early in life and mortality from each cause. Faster-growing males experienced lower non-hunting mortality as subadults and lower hunting mortality marginal on age. Females of all age classes did not display a strong association between their early-life growth rate and either mortality type. Our study does not provide evidence for a clear trade-off between early-life growth and mortality.

**Electronic supplementary material:**

The online version of this article (10.1007/s00442-020-04633-9) contains supplementary material, which is available to authorized users.

## Introduction

Harvesting acts as a strong selective pressure for early reproduction (Conover and Munch 2002; Festa-Bianchet 2003; Proaktor et al. 2007). High body growth rates allow individuals to reach the threshold size for reproduction early in life (Ricklefs 1969; Gadgil and Bossert 1970). As a consequence, fast early-life growth could be selected for in intensively hunted populations. However, fast early-life growth might be associated with some mortality costs. Following the principle of allocation (Cody 1966), fast early-life growth comes at the expense of other life-history traits such as somatic maintenance (Rollo 2002; Metcalfe and Monaghan 2003). An immediate natural mortality cost that may result from fast early-life growth can come in the form of reduced immune function in mammals (McDade 2005; but see Cheynel et al. 2019). Faster-growing individuals may thus experience higher natural mortality than slower-growing counterparts due to physiological costs associated with fast early-life growth. However, differences in individual quality in both resource acquisition and allocation may partially or completely mask trade-offs between life-history traits (van Noordwijk and de Jong 1986; Hamel et al. 2009; Wilson and Nussey 2009).

High-quality individuals (where quality is referred to as a positive covariation among performance traits that maximize lifetime reproductive success; see Wilson and Nussey 2009) exhibit secondary traits and behaviors that allow both high survival and high reproduction within their environmental context. Individuals of high quality are better able to acquire resources and thereby their probability of dying from natural causes is reduced compared to low-quality individuals (Bérubé et al. 1999; Blums et al. 2005). Therefore, high-quality individuals with fast early-life growth are expected to be those with lower natural mortality, and thus we expect a negative relationship between early-life growth and natural mortality for high-quality individuals. The resulting relationship between early-life growth and mortality may therefore be driven by a resource allocation trade-off and/or heterogeneity in individual quality.

The type of covariation among life-history traits is context dependent, with factors such as sex and age influencing their relationship. Predation risk modifies how populations seek out resources, for example by changing home range sizes, foraging time, or habitat selection (Creel and Christianson 2008). Behavioral effects of hunting can be stronger than those induced by non-human predators (see Proffitt et al. 2009 for an example of wolves and human predation on elk *Cervus elaphus*). Hunting may therefore influence how individuals acquire and allocate resources as well as the characteristics of a high-quality individual. For example, individuals that exhibit risky behavior and acquire more resources have higher early-life growth rates and are able to reproduce at a younger age than more cautious and slower-growing peers. However, when exposed to a high hunting pressure, bolder individuals may then face a higher probability of being harvested (Biro et al. 2006; Stamps 2007). Therefore, although faster early-life growth may be advantageous in some contexts, this faster growth schedule may come at an increased hunting risk. In the case of a hunted population, the highest-quality individuals are those able to acquire the highest amount of resources while also avoiding hunters. High-quality individuals therefore minimize predation risk when acquiring resources (Festa-Bianchet 1988 in bighorn sheep *Ovis canadensis*; Altendorf et al. 2001 in mule deer *Odocoileus hemionus*; Verdolin 2006 for a meta-analysis).

Changes to habitat use in response to hunting disturbance may differ between sexes (Saïd et al. 2012) and across age classes (Ciuti et al. 2012). Moreover, there is compelling evidence for differential allocation to early-life growth between males and females, resulting in different mortality costs for each sex. In polygynous species displaying strong sexual size dimorphism, males usually grow faster than females (e.g., red deer *Cervus elaphus* Clutton-Brock et al. 1982; but see Byers and Moodie 1990). We can thus expect mortality costs of growing fast to differ between sexes in species subjected to a strong sexual size dimorphism. The difference in natural mortality costs between sexes for fast early-life growth is expected to reflect the time to reach sexual maturity. The sex that reaches sexual maturity at a younger age is therefore expected to pay a cost at a younger age than the sex that displays a prolonged early-life growth. Thus, differences such as age and sex could influence the relationship between early-life growth rate and different mortality types (i.e., natural mortality vs. hunting mortality) in hunted populations.

The scarce empirical evidence available for a relationship between early-life growth and survival in harvested populations generally indicates that growing fast entails a cost, although there are notable exceptions (Table 1, Appendix S1). It is noteworthy that some studies have failed to detect a relationship between early-life growth and survival (e.g., Bergeron et al. 2008; Bonenfant et al. 2009), while others have found positive relationships (Chambellant et al. 2003; Beauplet et al. 2005; Nuñez et al. 2015). In these studies, high individual quality (with traits such as a heavy weight at birth) was strongly related to fast early-life growth and lower mortality rates. While investigating the potential effect of growing fast on survival in harvested populations, it is important to consider that most of the studies did not distinguish among the causes of mortality (Table 1, Appendix S1). Mortality from hunting and non-hunting causes were generally pooled as “overall mortality” (e.g., Loehr et al. 2007; Jorgensen and Holt 2013; but see Bonenfant et al. 2009). Moreover, all studies dealing with harvested populations only focused on one sex (Table 1, Appendix S1), preventing an assessment of between-sex differences (e.g., Robinson et al. 2006). A study linking early-life growth and age-specific mortality rates for individuals who experienced two types of mortality (natural and hunting) in males and females would allow further understanding of the mortality costs of fast early-life growth.

**Table 1:** Studies linking early-life growth rates to survival (non-exhaustive list)

Species	Order	References	Effect			
			Males	Females	Study type	Exploited
Bighorn sheep	Artiodactyla	Bonenfant et al. (2009)	(0)	NA	Field	Yes
*Ovis canadensis*						
Dall sheep	Artiodactyla	Loehr et al. (2007)	(−)	NA	Field	Yes
*Ovis dalli*						
Stone sheep	Artiodactyla	Douhard et al. (2016)	(−)	NA	Field	Yes
*Ovis dalli stonei*						
Alpine ibex	Artiodactyla	Toïgo et al. (2013)	(0)^a^	NA	Field	No
*Capra ibex ibex*						
Alpine ibex	Artiodactyla	Bergeron et al. (2008)	(0)	NA	Field	No
*Capra ibex ibex*						
Chamois	Artiodactyla	Bleu et al. (2014)	NA	(−)	Field	No
*Rupicapra rupicapra*						
Chamois	Artiodactyla	Corlatti et al. (2017)	(0)	(0)	Field	No
*Rupicapra rupicapra*						
Chamois	Artiodactyla	Corlatti et al. (2017)	(−)	( ±)^b^	Field	Yes
*Rupicapra rupicapra*						
European mouflon	Artiodactyla	Kavčić et al. (2019)	(−)	NA	Field	Yes
*Ovis orientalis*						
Subantarctic fur seal	Carnivora	Chambellant et al. (2003)	(+)	(0)	Field	No
*Arctocephalus tropicalis*						
Subantarctic fur seal	Carnivora	Beauplet et al. (2005)	(+)	(+)	Field	No
*Arctocephalus tropicalis*						
Three-spined stickleback	Gasterosteiformes	Lee et al. (2012)	(−)	(−)	Experimental	No
*Gasterosteus aculeatus*						
Speckled wood butterfly	Lepidoptera	Gotthard et al. (1994)	(−)	(−)	Experimental	No
*Pararge aegeria*						
Perch	Perciformes	Metcalfe and Monaghan (2003) and Craig (1980)	(−)	NA	Field	No
*Perca fluviatil*						
European plaice	Pleuronectiformes	Jorgensen and Holt (2013)	NA	(−)	Theoretical model	Yes
*Pleuronectes platessa*						
Rhesus Macaques	Primates	Nuñez et al. (2015)	( +)	( +)	Experimental	No
*Macaca mulatta*						
Wild type mice	Rodentia	Rollo (2002)	( − )	(−)	Experimental	No
*Muridae Mus*						
Norway rats	Rodentia	Rollo (2002)	(−)	(−)	Experimental	No
*Rattus norvegicus*						
Tasmanian snow skinks	Squamata	Olsson and Shine (2002)	(−)	(−)	Experimental	No
*Niveoscincus mircolepidotus*						

We reported if early-life growth had a positive ( +), negative (−), no (0), or untested (NA) effect on survival. The literature survey was performed using ISI Web of Science and Google Scholar using combinations of the keywords “early-life growth rate,” “juvenile growth rate”, “trade-off”, “survival”, “mortality”, “growth–lifespan trade-off”, “growth–survival trade-off”, and “growth–mortality trade-off.” In addition, the bibliographies of relevant papers were used to search for studies to include in the review. These terms were kept broad as the relationship between early-life growth and survival can be analyzed in a study but not be its focus. Only studies performed on animal species were retained. The search was conducted in February 2020. For more precise information from each paper detailing the trade-off, see Appendix S1

^a^Early-life growth was not related to survival until late life, when early horn growth incurred a survival cost

^b^The culling regime and hunter preference determined survival patterns in the two harvested populations

Taking advantage of a unique long-term monitoring study of an intensively hunted population of wild boar (*Sus scrofa*), we aimed to assess both whether early-life growth is associated with subsequent mortality and whether sex and mortality cause influenced this potential association. We first looked for the relationship between early-life growth rate and overall mortality in both sexes. Then, we explored the relationship between early-life growth rate and cause-specific mortality in both sexes. In a highly dimorphic and polygynous species such as wild boar (Toïgo et al. 2008), we could expect sex-specific differences in the strength of the relationship between early-life growth rate and natural mortality. In particular, wild boar males and females start growing at the same rate, but females stop growing well before males (Gaillard et al.^1992^). Also, wild boar females exhibit a lower threshold body mass for reproduction than other species of large herbivores (Servanty et al. 2009). We thus expect females to pay a natural mortality cost at a younger age than males, which display a prolonged growth period. As the hunting pressure is strong in this system, we expect high hunting mortality for individuals regardless of sex, age, or early-life growth rate.

## Materials and methods

### Study area and data collection

**Fig. 1:**
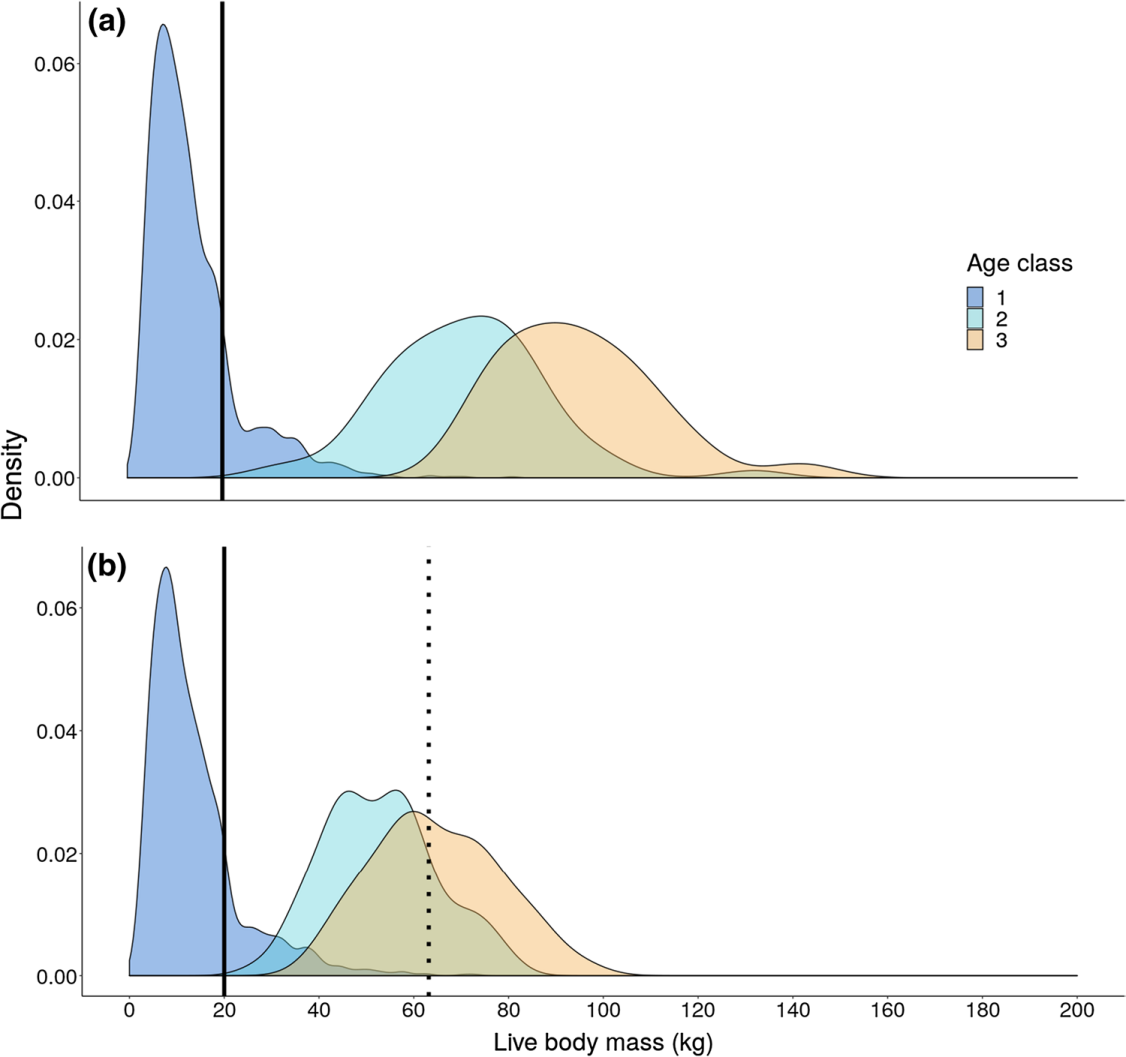
The distribution (displayed as kernel density estimates) of (**a**) male and (**b**) female body mass of individual wild boar in relation to age class. Age class one corresponds to birth to 1 year of age (i.e., juveniles), age class two corresponds to one to 2 years of age (i.e., subadults), and age class three corresponds to individuals older than 2 years of age (i.e., adults). Individuals included in the analysis were captured for the first time in their first year of life (age class one) and were captured at least twice with a live body mass measurement of or below 20 kg (this body mass threshold is indicated by the black solid lines) to estimate their early-life growth rate. Females at or above 63 kg (i.e., with a dressed body mass at or above 50 kg, see Gamelon et al. 2012) were protected by a hunting restriction (black dotted line)

We analyzed data collected from a long-term study of a hunted wild boar population located in the Châteauvillain-Arc-en-Barrois forest. The 11,000 ha forest is located in north-eastern France (45°02′; 4°55′ E) and is characterized by a climate intermediate between continental and oceanic. Capture-mark-recapture data were collected annually between March and September from 1983 to 2017. Individuals weighing less than 20 kg (i.e., juveniles < 1 year of age) were captured using traps, marked, and released (Fig. 1). Sex, date, and body mass to the nearest 0.1 kg were recorded for each individual. Individuals were recaptured after at least 1 week has passed since the previous capture event. Therefore, all body mass measurements were more than 7 days apart. These data were collected for 516 males and 475 females.

Starvation, disease, and vehicle collisions accounted for most non-hunting mortality in this population. As only 5 out of 992 individuals died from vehicle collisions in our dataset, non-hunting mortality is a good proxy of natural mortality. Hunting was the main source of mortality (Gamelon et al. 2011). While wild boars have been growing in numbers appreciably throughout Europe during the last decades, they are managed at a local scale. At Châteauvillain-Arc-en-Barrois, wild boars are harvested by drive hunts between October and February each year during the study period. Each weekend during the hunting period, drive hunts are organized. Ambush hunters are posted around a given area and wait for wild boars startled by beaters and dogs (Saïd 2012; Vajas et al. 2020). Wild boars are killed when they are flushed out of the hunted plot. In that respect, hunting is not oriented toward any specific age or body mass class. However, large females are protected from hunters who have to pay a penalty when shooting females over 50 kg (dressed body mass, Gamelon et al. 2012), which corresponds to about 63 kg live body mass (Fig. 1b). Such a hunting regulation did not exist for males. Due to the unique life history of wild boar, the protected threshold size for hunting (63 kg) is reached as early as 2 years of age by most females (see Fig. 1). Thanks to the social structure of wild boar as well as strong phenotypic differences between sexes and ages, hunters can easily assess sex and approximate body mass, and thus avoid shooting the largest females (Gamelon et al. 2012). Indeed, wild boars live in matrilineal social groups and males are solitary (Kaminski et al. 2005), making the determination of sex straightforward. Also, a female group is led by a large sow (generally weighing more than 63 kg), followed by juveniles that are markedly smaller. They are striped until 4 months of age and then wear a reddish coat until they reach about 30 kg. This makes the determination of body mass straightforward. As a consequence, because of the high hunting pressure and the hunting restriction on large females, a large proportion of individuals less than 1 year of age is shot in that population (between 60 and 80%, see Fig. 1 in Gamelon et al. 2011). Live and dressed body mass (recorded after removing the digestive system, heart, lungs, liver, reproductive tract, and blood) as well as sex was recorded for each hunted wild boar. When live body mass information was not collected, the dressed body mass was converted to live body mass using the established relationship between these metrics (see Gamelon et al. 2017). Emigration was not expected to contribute to non-hunting mortality as wild boar emigration is very low (except for subadult males, see Truvé and Lemel 2003; Keuling et al. 2010). Hereafter, we define year in relation to the hunting season, from October 1 in a given year to October 1 the next year.

### Estimating early-life growth rate

Wild boar included in the analysis had at least two recorded live body mass measurements below 20 kg as juveniles (Fig. 1), in the first few months of life. The number of times an individual was captured was not strongly related to its early-life growth rate (Pearson correlation coefficient between the number of captures and early-life growth rate = 0.19, *p* value ≤ 0.01). Although statistically significant, the relationship was weak because only 4% of the variation in the early-life growth rate observed across wild boars was accounted for by differences in the number of times these individuals were captured. As growth rates are linear in the first 6 months of life in wild boar (Gaillard et al. 1992), we estimated the early-life growth rate (*G*_*i*_) of each individual as:

$$ G_{i} { = }\frac{{W_{n } - W_{{1}} }}{{T_{{{\text{elapsed}}}} }}, $$

where *W*_*n*_ is the last recorded body mass (in grams) at either last recapture or recovery (at or below a live body mass of 20 kg), *W*_1_ is the body mass at first capture (in grams) and *T*_elpased_ is the number of days elapsed between the two measurements. We checked the assumption that early-life growth rates are effectively linear by comparing this method to a second method that used the average of growth rates early in life (see Appendix S2), which had a weaker assumption of linearity. It is noteworthy that the two methods produced highly similar early-life growth rate estimates (see Appendix S2, Fig. S2).

### Estimating overall mortality

We estimated the overall mortality probability using capture–mark–recapture–recovery (CMRR) analysis (Lebreton et al. 2009). Noticeably, emigration is very low for this species, as females are sedentary, except for subadult males that leave matrilineal groups and disperse to live alone (Truvé and Lemel 2003; Keuling et al. 2010). Overall mortality is thus “apparent” and includes both the probability of dying and the probability of dispersing/emigrating for subadult males, whereas it mostly represents true mortality for females and males of other ages. Analyses were performed for males and females separately. First, we tested the goodness of fit (GOF; Pradel et al. 2005) of these models using U-CARE (Choquet et al. 2009). As mortality rates are slightly age specific in wild boar (Gamelon et al. 2011; Toïgo et al. 2008), we distinguished three age classes: juveniles (less than 1 year olds), subadults (between 1 and 2 years old), and adults (more than 2 years old) (Fig. 1). We did not look for further age dependence in adult wild boar because the oldest male was only 5 years of age and the oldest female was 8 years of age in our dataset, likely as a consequence of the intensive hunting pressure (Toïgo et al. 2008). We explored whether overall mortality differed among age classes. For the analysis, we define *p* as the probability of live individuals to be recaptured (i.e., the probability for an individual to be recaptured in a trap), and *r* as the probability of individuals shot by hunters to be recovered (i.e., the probability for an individual to be recovered by the hunters when killed). As capture and recovery protocols were kept constant throughout the study period (Gamelon et al. 2011), *p* and *r* were assumed to be constant over time, as done in Gamelon et al. (2011, 2012). Consistent with previous studies for this population, *p* was generally low (see results). This indicates that an individual captured at year *t* has a low probability to be recaptured at year *t* + *1*. There was no evidence for contrasting recapture rates between ages, which are consistently very low. To test the assumption of a constant recapture probability *p* throughout the study period, we compared mortality estimates with constant and time-varying *p*. Models with a time-varying *p* struggled to produce estimates for *p* due to low sample size. However, mortality estimates for models with constant and time-varying recapture rates *p* were highly similar for all models (results not shown here). We therefore did not consider different recapture rates over years and among age classes. On the contrary, *r* was very high, approaching 1 (see results). Such a high recovery rate was due to the involvement of the French National Agency for Wildlife and Hunting (OFB) that collected all the wild boar shot in cooperation with hunters. Thus, most of the individuals killed by hunters were then collected and identified if they were previously marked. We therefore did not expect recovery rates to differ over years and among age classes.

We used the Akaike information criterion corrected for small sample size (AICc, Burnham and Anderson 2002) to compare the candidate models used to assess whether overall mortality differed among age classes. When AICc values of two competing models were within two units, we retained the simplest model (i.e.. the model with the fewest parameters) to satisfy parsimony rules.

### Estimating cause-specific mortality

CMRR analyses (Lebreton et al. 2009) were used to estimate cause-specific mortality by performing the joint analysis of recaptures of live individuals and recoveries of hunted individuals (Schaub and Pradel 2004). Individuals were considered to be in one of four states: (1) “alive”, (2) “dead by hunting”, (3) “dead by non-hunting causes”, and (4) “already dead”, the absorbing state. States (3) and (4) were not observable because information was only available for individuals that were shot by hunters. All individuals in states (2) and (3) at year *t* moved to the absorbing state (4) at *t* + *1* (see Appendix S3 for event matrices). Thus, hunting mortality corresponded to the transition from the state “alive” (1) at year *t* to the state “dead by hunting” (2) at year *t* + *1* and non-hunting mortality corresponded to the transition from the state “alive” (1) at year *t* to the state “dead by non-hunting causes” (3) at year *t* + *1* (see Appendix S3 and Gamelon et al. 2011 for transition and event matrices). As wild boar are sedentary, non-hunting mortality represents the true probability of dying from non-hunting causes, except for subadult males for which non-hunting mortality represents both the probability of dying from non-hunting causes and the probability of dispersing/emigrating. To ensure all probabilities fell within the range [0–1], we used a generalized (multinomial) logit-link function. As done for overall mortality, *p* and *r* were assumed to be constant over time and we explored whether cause-specific mortalities differed among age classes using AICc for model comparison.

### Linking early-life growth rate and mortality

To explore the effect of early-life growth on overall mortality, we included growth rate as an individual covariate to the best model with the selected age structure. Early-life growth rate was thus treated as a continuum in the analyses and entered as a continuous variable. As all age classes exhibit the same overall mortality for males (see results), we tested an effect of early-life growth rate on overall mortality with all ages pooled together. For females, age class 1 (juveniles) has a different overall mortality than older individuals (see results). Similarly, we assessed the effect of early-life growth on both hunting and non-hunting mortality by including the growth rate as a continuous individual covariate in the selected model that distinguished between the causes of mortality.

In addition to considering growth rate as a continuous variable, we considered it as a categorical variable. We thus split the male and female datasets into 15 classes of early-life growth rates, each class including approximately 32 individuals (see Appendix S4 for minimum and maximum early-life growth rates for each categorical class in g/day). We then estimated overall mortality and cause-specific mortality for each class of early-life growth rate by entering growth rate as a categorical variable. To further explore the age-specific mortality of individuals that experienced negative early-life growth rates, models with mortality estimated for individuals with either a negative or a positive growth rate were used. Early-life growth rate was included as a categorical variable to estimate age-specific mortalities for individuals in one class that had a negative to zero early-life growth or greater than zero early-life growth rate in a separate class.

All analyses were performed using the program E-SURGE (Choquet et al. 2009).

## Results

### Early-life growth rate

**Fig. 2:**
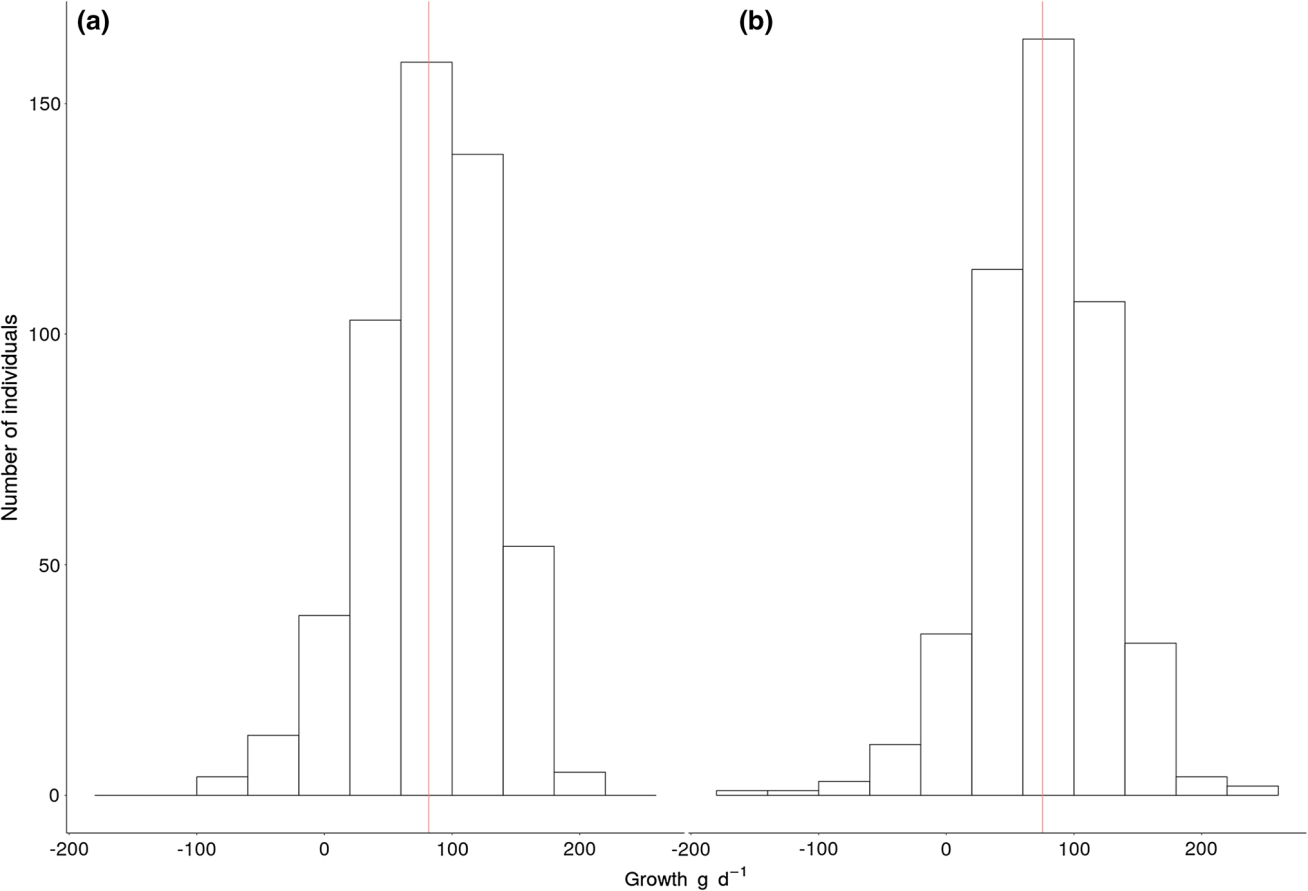
Distribution of early-life growth rates (i.e., for individuals weighing up to 20 kg) for (**a**) male and (**b**) female wild boar at Châteauvillain-Arc-en-Barrois. Red vertical lines indicate the average growth rate for each sex

The average early-life growth rate was 82.67 g/day (max = 214.29 g/day, min = − 86.21 g/day) for males and 76.29 g/day (max = 226.19 g/day, min = − 170.00 g/day) for females (Fig. 2). It is notable that some individuals had negative growth rates.

### Linking early-life growth rate to overall mortality

**Fig. 3:**
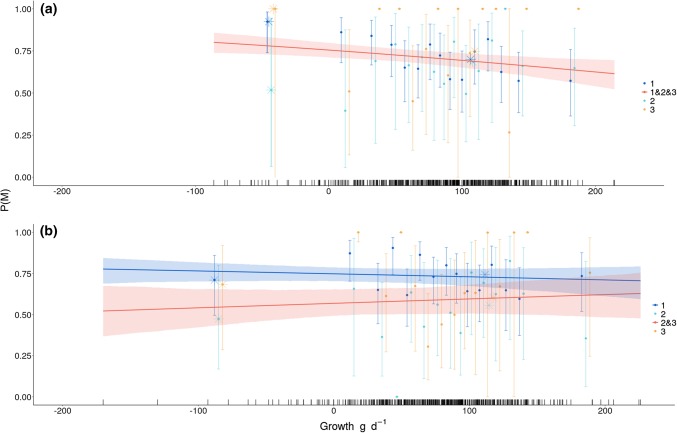
Age-specific (1, 2, 3) overall mortality P(M) as a function of early-life growth rate (in g/day) for (**a**) male and (**b**) female wild boar in Châteauvillain-Arc-en-Barrois. The points depict the mortality estimates and associated 95% confidence intervals for each class of early-life growth rates from models with early-life growth rate included as a categorical variable. The lines show estimates from the selected models with early-life growth rate as a continuous individual covariate (see Table 2A) and associated 95% confidence intervals. The rug plot shows the respective distributions of early-life growth rates for each sex. The stars depict age-specific mortality estimates from models with either negative to zero early-life growth rates or positive early-life growth rates. The estimates from the categorical models are plotted against the median value in the range of early-life growth rates for a given bin

The GOF test did not detect any lack of fit (global test for males: *P* = 0.20, *df* = 62; for females: *P* = 0.20, *df* = 79). For males, the selected model without including growth indicated constant rates of overall mortality across age classes, with an estimated overall mortality rate of 0.71 (SE: 0.02) (Table 2A, males, M1). From this model, the recapture probability was 0.27 (SE: 0.03) and the recovery rate was 0.72 (SE: 0.02). We found no evidence that mortality differs among age classes (Table 2A, males, M5, ΔAICc of 3.22), which suggests similar mortality rates across age classes. Adding the growth rate as an individual covariate to the selected model, we found that early-life growth rate and overall mortality were negatively associated, indicating that fast-growing males had lower mortality marginal on age (Fig. 3a). From the models with negative vs. positive early-life growth as a categorical variable, the youngest and oldest males with negative early-life growth rates had the highest probability of dying (juvenile *M* = 0.92, SE: 0.05; adult *M* = 0.99, SE: 0.02), whereas subadults with negative early-life growth rates had the lowest (*M* = 0.52, SE: 0.35; stars, Fig. 3a). Juvenile and adult males with positive growth rates had a lower probability of dying across age classes (juvenile *M* = 0.70, SE: 0.02; subadult *M* = 0.68, SE: 0.05; adult *M* = 0.75, SE: 0.07) than males with negative early-life growth rates.

**Table 2:** Model selection for overall mortality (A) and cause-specific mortality (B) in wild boar

Model name	Model notation	*Np*	Biological meaning	AICc for males	AICc for females
A					
M1	M(1&2&3)	3	Same overall mortality for age classes 1, 2 and 3	**1512.43**	1548.44
M2	M(1&3, 2)	4	Same overall mortality for age classes 1 and 3; different for age class 2	1513.92	1540.30
M3	M(1&2, 3)	4	Same overall mortality for age classes 1 and 2; different for age class 3	1514.01	1547.80
M4	M(1, 2&3)	4	Same overall mortality for age classes 2 and 3; different for age class 1	1514.41	**1535.85**
M5	M(1, 2, 3)	5	Different overall mortalities for age classes 1, 2, and 3	1515.65	1537.30
B					
M1	Mh(1&2&3), Mn(1&3, 2)	5	The same hunting mortalities for age classes 1, 2, and 3; different non-hunting mortalities for age class 2 than 1 and 3	**1503.49**	1549.67
M2	Mh(1&2&3), Mn(1,2,3)	6	The same hunting mortalities for age classes 1, 2, and 3; different non-hunting mortalities for age classes 1, 2, and 3	1504.77	1551.20
M3	Mh(1&2, 3), Mn(1, 2, 3)	7	The same hunting mortalities for age classes 1 and 2, and a different hunting mortality for age class 3; different non-hunting mortalities for age classes 1, 2, and 3	1505.05	1551.31
M4	Mh(1, 2&3), Mn(1, 2, 3)	7	The same hunting mortalities for age classes 2 and 3, and a different hunting mortality for age class 1; different non-hunting mortalities for age classes 1, 2, and 3	1505.06	1539.77
M5	Mh(1&2, 3), Mn(1&3, 2)	6	Different hunting mortalities for age class 3 than age classes 1 and 2; different non-hunting mortalities for age class 2 than age classes 1 and 3	1505.42	1551.93
M6	Mh(1&3, 2), Mn(1&3, 2)	6	The same hunting mortalities for age classes 1 and 3, and a different hunting mortality for age class 2; different non-hunting mortality for age class 2 than 1 and 3	1505.43	1541.89
M7	Mh(1, 2&3), Mn(1&3, 2)	6	The same hunting mortalities for age classes 2 and 3, and a different hunting mortality for age class 1; different non-hunting mortality for age class 2 than 1 and 3	1505.80	1539.79
M8	Mh(1, 2, 3), Mn(1, 2, 3)	8	Different hunting mortalities for age classes 1, 2, and 3; different non-hunting mortalities for age classes 1, 2, and 3	1506.42	1541.23
M9	Mh(1&3, 2), Mn(1, 2, 3)	7	The same hunting mortalities for age classes 1 and 3 and a different hunting mortality for age class 2; A different non-hunting mortality for age classes 1, 2, and 3	1506.54	1543.73
M10	Mh(1, 2, 3), Mn(1&3, 2)	7	Different hunting mortalities for age classes 1, 2 and 3; A different non-hunting mortality for age class 2 than for 1 and 3	1506.80	1541.36
M11	Mh(1&2, 3), Mn(1, 2&3)	6	The same hunting mortalities for age classes 1 and 2, a different hunting mortality for age class 3; A different non-hunting mortality for age classes 2 and 3 than 1	1510.27	1552.23
M12	Mh(1&2&3), Mn(1, 2&3)	5	The same hunting mortalities for age classes 1, 2, and 3; A different non-hunting mortality for age class 1 than for 2 and 3	1510.29	1550.42
M13	Mh(1, 2, 3), Mn(1, 2&3)	7	Different hunting mortalities for age classes 1, 2 and 3; The same non-hunting mortality for age classes 2 and 3 and a different non-hunting mortality for age class 1	1511.93	1541.23
M14	Mh(1, 2&3), Mn(1, 2&3)	6	Different hunting mortalities for age classes 2 and 3 than age class 1; The same non-hunting mortality for age classes 2 and 3 and a different non-hunting mortality for age class 1	1512.24	1537.76
M15	Mh(1&2&3), Mn(1&2&3)	4	The same hunting mortalities for age classes 1, 2, and 3; The same non-hunting mortalities for age classes 1, 2, and 3	1512.43	1548.43
M16	Mh(1&3, 2), Mn(1, 2&3)	6	The same hunting mortalities for age classes 1 and 3 and a different hunting mortality for age class 2. The same non-hunting mortality for age classes 2 and 3 and a different non-hunting mortality for age class 1	1512.60	1543.73
M17	Mh(1&3, 2), Mn(1&2&3)	5	The same hunting mortalities for age classes 1 and 3 and a different hunting mortality for age class 2. The same non-hunting mortality for age classes 1, 2, and 3	1513.07	1541.89
M18	Mh(1&2&3), Mn(1&2, 3)	5	The same hunting mortalities for age classes 1, 2, and 3. Different non-hunting mortality for age classes 1 and 2 than age class 3	1513.09	1549.65
M19	Mh(1&2, 3), Mn(1&2, 3)	6	Different hunting mortalities for age classes 1 and 2 than age class 3. Different non-hunting mortality for age classes 1 and 2 than age class 3	1514.37	1549.56
M20	Mh(1, 2, 3), Mn(1&2&3)	6	Different hunting mortalities for age classes 1, 2, and 3. The same non-hunting mortality for age classes 1, 2, and 3	1514.51	1539.22
M21	Mh(1&3, 2), Mn(1&2, 3)	6	The same hunting mortalities for age classes 1 and 3 and a different hunting mortality for age class 2. The same non-hunting mortality for age classes 1 and 2 and a different non-hunting mortality for age class 3	1514.92	1543.74
M22	Mh(1&2, 3), Mn(1&2&3)	5	A different hunting mortality for age class 3 than for age classes 1 and 2. The same non-hunting mortality for age classes 1, 2, and 3	1515.13	1549.86
M23	Mh(1, 2&3), Mn(1&2&3)	5	A different hunting mortality for age class 1 than 2 and 3. The same non-hunting mortality for age classes 1, 2, and 3	1515.32	**1537.79**
M24	Mh(1, 2, 3), Mn(1&2, 3)	7	Different hunting mortalities for age classes 1, 2, and 3. The same non-hunting mortality for age classes 1 and 2 and a different non-hunting mortality for age class 3	1516.48	1541.23
M25	Mh(1, 2&3), Mn(1&2, 3)	6	A different hunting mortality for age class 1 than for age classes 2 and 3. The same non-hunting mortality for age classes 1 and 2 and a different non-hunting mortality for age class 3	1516.48	1539.80

Displayed are models relating age classes to overall mortality (*M*), hunting mortality (*Mh*), and non-hunting mortality (*Mn*). Age classes are denoted as 1 for juveniles, 2 for subadults, and 3 for adults. Pooled age classes are indicated with ‘&’ between them. ‘Np’ indicates the number of biological parameters. ‘Biological meaning’ explains the ‘Model notation’ in biologically relevant terms. When two models had close AICc values (ΔAICc < 2), the most parsimonious model was selected. The selected models are indicated in bold

For females, the selected model revealed a higher overall mortality for juveniles than for subadults and adults (Table 2A, females, M3). The mortality estimates were 0.74 (SE: 0.02) for juvenile and 0.58 (SE: 0.03) for older (i.e., subadult and adult) females from the best model without including the growth rate covariate. The recapture probability was 0.43 (SE: 0.04) and the recovery rate was 0.71 (SE: 0.02). This model performed slightly better than the model that included age-specific mortality rates (Table 2A, females, M5, ΔAICc = 1.45), and much better than a model with constant mortality across age classes (Table 2A, Females, M1, ΔAICc = 12.59). Adding the growth rate as an individual covariate to the selected model, early life-growth rate was weakly related to overall mortality rate across age classes (Fig. 3b). From the models with early-life growth as a categorical variable that was either negative or positive, juveniles and adults with negative early-life growth rates had similarly high probabilities of experiencing mortality (stars, Fig. 3b). Females with positive early-life growth rates had similar mortality probabilities (juvenile *M* = 0.74, SE: 0.02; subadult *M* = 0.56, SE: 0.05; adult *M* = 0.60, SE: 0.06) as females with negative early-life growth rates (juvenile *M* = 0.71, SE: 0.10; subadult *M* = 0.47, SE: 0.19; adult *M* = 0.68, SE: 0.19) across age classes.

### Linking early-life growth rate to cause-specific mortality

For males, the best cause-specific mortality model included a high hunting mortality *Mh* of 0.59 (SE: 0.02) for all age classes (Table 2B, males, M1). Non-hunting mortality *Mn* was 0.07 (SE: 0.02) for juveniles and adults, and 0.38 (SE: 0.08) for subadults (Table 2B, males, M1). The recapture probability was 0.24 (SE: 0.03) and the recovery rate was 0.91 (SE: 0.02). We found no support for constancy in both non-hunting mortality and hunting mortality across age classes (Table 2B, males, M15, ΔAICc = 8.94).

**Fig. 4:**
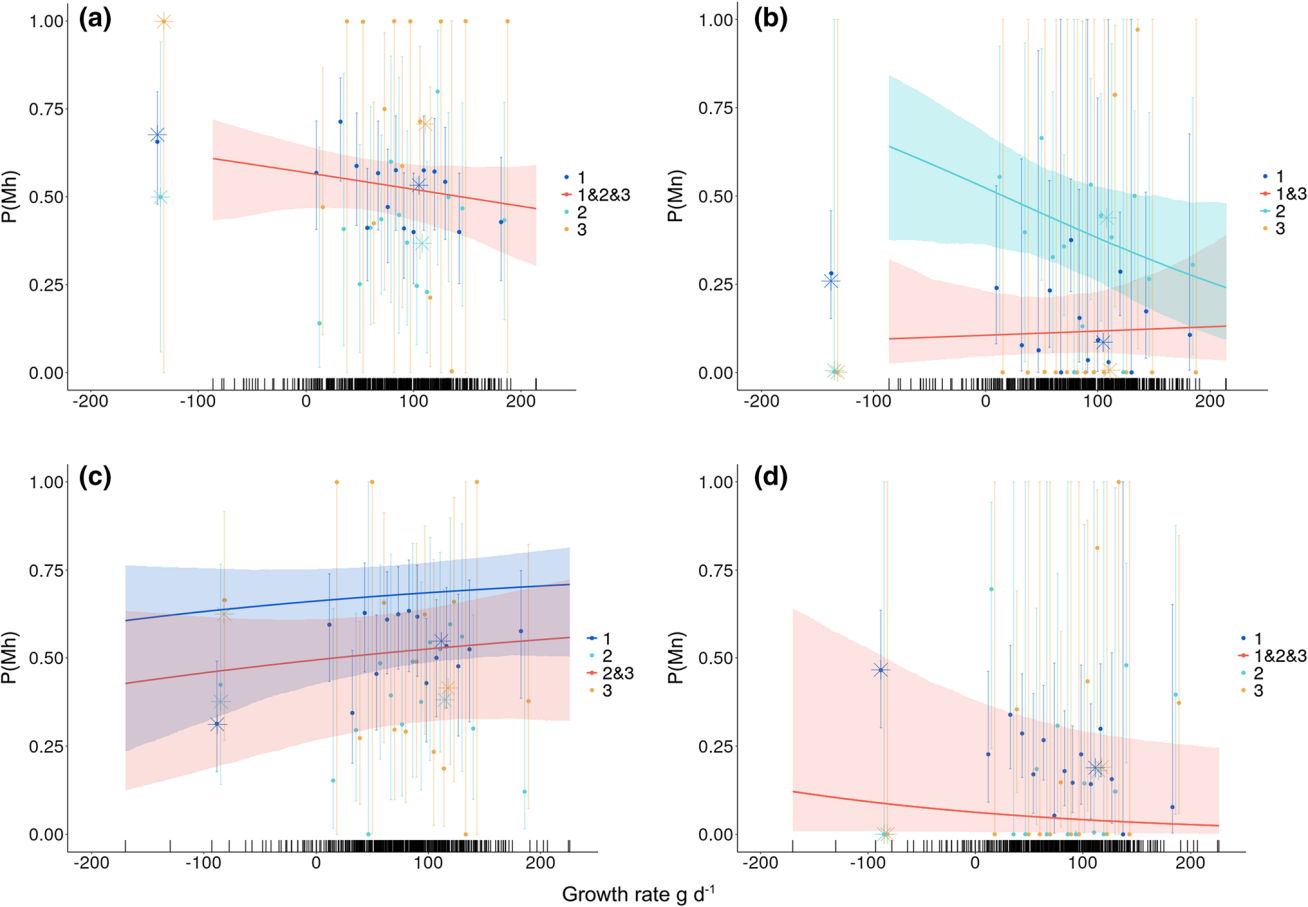
Age-specific hunting mortality (**a** and **c**; P(Mh)) and non-hunting mortality (**b**, and **d**; P(Mn)) as a function of early-life growth rate (in g/day) for (**a** and **b**) male and (**c** and **d**) female wild boar in Châteauvillain-Arc-en-Barrois. The points depict the mortality estimates and 95% confidence intervals from a model for each class of early-life growth rates included as a categorical variable. The lines correspond to mortality estimates for each age class included in the selected models with early-life growth rate as a continuous variable (see Table 2B) and associated 95% confidence intervals. The rug plot shows the distributions of early-life growth rates for each sex. The stars depict age-specific mortality estimates from models with either negative to zero early-life growth rates or positive early-life growth rates. The estimates from the categorical models are plotted against the median value in the range of early-life growth rates for a given bin

When the individual covariate representing early-life growth was added to the best model, we found a negative association between early-life growth rate and hunting mortality for males (Fig. 4a, red curve), indicating that fast-growing males early in life had lower hunting mortality rates at all ages than slower-growing counterparts. Similarly, we found that faster-growing males had a lower non-hunting mortality rate as subadults than slower-growing individuals (Fig. 4b, light blue line). However, for juvenile and adult males, there was no evidence of a relationship between early-life growth rate and non-hunting-related mortality (Fig. 4b, red line). Among individuals exhibiting a negative early-life growth rate, adults faced the highest probability of being hunted (juvenile *Mh* = 0.68, SE: 0.11, subadult *Mh* = 0.50, SE: 0.36, adult *Mh* = 0.99, SE: 0.06; stars, Fig. 4a), while juveniles had the highest probability of dying from non-hunting mortality (juvenile *Mn* = 0.26, SE: 0.11, subadult *Mn* ≤ 0.01, SE: 0.11, adult *Mn *≤ 0.01, SE: 0.05; stars, Fig. 4b). Males with positive early-life growth rates had a lower probability of being hunted across age classes than males with negative early-life growth rates. Among individuals exhibiting positive growth rates, juveniles *Mn* = 0.09, SE: 0.07) and adults (*Mn *≤ 0.01, SE: < 0.01) had a very low probability of dying from non-hunting mortality, while subadults had a higher probability (*Mn* = 0.44, SE: 0.09). Males with positive early-life growth rates always had a lower probability of being hunted (juvenile *Mh* = 0.53, SE: 0.06, subadult *Mh* = 0.37, SE: 0.06, adult *Mh* = 0.71, SE: 0.08) across age classes than males with negative early-life growth rates.

For females, two models had nearly the same AICc values (Table 2B, females, M14 and M23, ΔAICc = 0.03) for cause-specific mortality. The selected model (Table 2B, females, M22), chosen following the rules of parsimony, indicated that hunting mortality *Mh* was 0.73 (SE: 0.13) for juveniles and 0.56 (SE: 0.18) for older females (i.e., subadults and adults). The non-hunting mortality (*Mn*) estimate for all females was 0.01 (SE: 0.11). The recapture probability was 0.43 (SE: 0.04) and the recovery rate 0.73 (SE: 0.15). The models including constant hunting and non-hunting mortality rates across age classes performed very poorly (Table 2B, Females, M15, ΔAICc = 14.01).

When the individual covariate representing early-life growth was added to the best model, we found that early-life growth rate was weakly related to both hunting and non-hunting-related mortalities. Thus, hunting mortality (Fig. 4c, dark blue and red lines) and non-hunting mortality (Fig. 4d, red line) did not appear to strongly depend on early-life growth rate (see Appendix S5 for slope and intercept estimates of the selected models on either the logit scale (for overall mortality) or generalized logit scale (for cause-specific mortality)). Females with a negative early-life growth rate were most likely to die from hunting as adults (*Mh* = 0.63, SE: 0.20, stars, Fig. 4c) and experienced non-hunting mortality as juveniles (*Mn* = 0.47, SE: 0.09; stars, Fig. 4d). Subadults (*Mn *≤ 0.01, SE: < 0.01) and adults (*Mn *≤ 0.01, SE: < 0.01) with negative early-life growth rates were very unlikely to die from non-hunting mortality. Females with positive early-life growth rates across age classes (juvenile *Mh* = 0.55, SE: 0.02, subadult *Mh* = 0.38, SE: 0.05, adult *Mh* = 0.41, SE: 0.06) had a higher hunting mortality probability than juvenile (*Mh* = 0.30, SE: 0.08) and subadult (*Mh* = 0.38, SE: 0.18) females with negative early-life growth rates. Females with positive growth rates across age classes had a very low probability of dying from non-hunting mortality (juvenile *Mn* = 0.19, SE: 0.02, subadult *Mn* = 0.18, SE: 0.07, adult *Mn* = 0.19, SE: 0.06) compared to juveniles with negative early-life growth rates.

## Discussion

Our approach is unique among studies linking early-life growth to mortality in harvested populations because it accounts for possible confounding effects of age, sex, and cause-specific mortality (i.e., non-hunting vs. hunting mortality). Classical approaches would have only tested for a relationship between early-life growth rate and overall mortality in both sexes. If we had only followed this approach without splitting mortality into its causes, we would have simply found that the fastest-growing males experienced lower overall mortality than their slower-growing counterparts (see Fig. 3a). However, in our population, hunting mortality accounted for most of the overall mortality compared to non-hunting mortality. Consequently, overall mortality models usually fitted in survival analyses of hunted vertebrate populations do not accurately depict how age-specific hunting versus non-hunting mortality is related to other life-history traits (Lebreton 2005; but see Schaub and Pradel 2004; Brodie et al. 2013; or Koons et al. 2014 who used cause-specific mortality models to assess different natural and human-related sources of mortality). Here, from the cause-specific models, we show that male juveniles and adults as well as females of all age classes display a very weak relationship between early-life growth rate and non-hunting-related mortality. Indeed, only the non-hunting mortality of subadult males was strongly related to early-life growth rate. In particular, slow-growing males exhibited the highest non-hunting mortality at age two. At this age, they disperse from their natal area and face increased mortality risks (Truvé and Lemel 2003). It should be noted that non-hunting-related mortality includes emigration. Therefore, the strong negative relationship between male subadult non-hunting-related mortality and early-life growth rate may be due to slow-growing males being more likely to die of non-hunting-related causes as subadults. Alternatively, males that grow slowly early in life may be more likely to disperse as subadults, and likely to acquire more resources. Splitting mortality into its causes is thus recommended to gain an understanding of the underlying mechanisms shaping the covariation between life-history traits when mortality can be mostly attributable to one cause (e.g., harvesting).

In addition, for males, hunting probability was negatively related to early-life growth rate (Fig. 4a). Further, from the models with negative and positive early-life growth rates, males with positive early-life growth rates had a lower probability of being hunted than those with a negative early-life growth rate for every age class. Therefore, we found that faster-growing males were less likely to be hunted than slower-growing individuals. This provides support for the hypothesis that males with high growth rates are also more able to evade being hunted, and are possibly of higher quality (similar to Festa-Bianchet 1988; Altendorf et al. 2001). However, we did not find strong evidence of a relationship between early-life growth rate and hunting mortality for females. Females that grew quickly had a slightly higher probability to be hunted than females that grew slowly (Fig. 4c). We therefore found no detectable evidence that individual ability to grow quickly early in life reduced hunting probability for females (i.e., faster-growing individuals had a lower probability of being hunted).

Some studies have reported a positive relationship between early-life growth and mortality (see Table 1). Our expectation in this population characterized by a high hunting pressure was that fast-growing females, in addition to allocating a large amount of resources to growth, would reach the threshold body mass to reproduce earlier than slower growing juveniles. We expected that earlier reproduction would then lead to an increase in non-hunting-related mortality costs. Indeed, fast-growing females consistently reach sexual maturity earlier than slower growing counterparts in most vertebrate species (e.g., Enberg et al. 2012 in fish; Flom et al. 2017 in humans). In the studied population, females only need to reach about 37% of their adult body mass to reproduce for the first time (Servanty et al. 2009) within their first year of life (Gamelon et al. 2011). We did not find evidence of a positive relationship between early-life growth and non-hunting mortality in females. As fast early-life growth did not increase the probability that wild boar experienced non-hunting mortality, we did not find evidence that fast early-life growth leads to higher non-hunting mortality. Note, however, that because of the high hunting pressure in this system, we assessed the costs of fast early-life growth at young ages. We tested for potential negative effects of fast growth rates on mortality at ages 0–1, 2, and 3 or more, whereas growth costs might occur much later in life. Indeed, in response to the high hunting pressure, only a few individuals were likely to die from non-hunting causes during adulthood, which explains the large confidence intervals in the estimates of non-hunting mortality of adults (Fig. 4). Also, we did not find a strong negative relationship between early-life growth and non-hunting mortality, so our results did not support the individual quality hypothesis (e.g., faster-growing individuals are less likely to die of starvation or disease). Most previous studies dealing with harvested populations did not distinguish among causes of mortality and generally did not explore such relationships between early-life growth and mortality in both sexes. Our study proves that disentangling mortality causes is important when the hunting pressure is strong in a population, as males and females can exhibit different responses.

There is increasing evidence that human exploitation induces rapid evolutionary changes in populations, which results in shortening the time between birth and first reproduction. While effects of harvest-induced changes to early-life growth rates are well documented in fisheries (Law 2000; Dunlop et al. 2009; Enberg et al. 2011), they remain largely unexplored in hunted mammals (see Table 1). This distinction is important as fish are indeterminate growers, and experience more flexibility in the age/size at maturity and therefore have a much greater variability in the individual relationship linking body size and reproduction than mammals. In many species, there is extensive evidence that a strong harvesting pressure can increase body growth rates, which allows reaching the threshold body size/mass for reproduction earlier (see Kuparinen and Festa-Bianchet 2017 for a review of evolutionary effects of harvesting). Noticeably, individuals might simply reproduce at smaller sizes, with unaffected body growth rates. Previous work on wild boar linked a high hunting pressure with a lower threshold body mass for reproduction and earlier birth dates, which stimulate high reproductive rates within the first year of life (Servanty et al. 2009; Gamelon et al. 2011). Thus, it is likely that harvest-induced selection in wild boar has resulted in a reduction of the size threshold for reproduction rather than an increase of early-life growth rate. Here, we were not able to demonstrate that early-life growth rate was linked to non-hunting mortality, rather we observed a weak or null relationship between these life-history traits (except in the case of subadult males). In particular, we found no evidence that fast-growing females that reach the mass/size threshold for reproduction at younger ages exhibit higher mortality costs than slow-growing individuals. However, males that grew quickly also were less likely to be hunted, indicating that heterogeneity in individual quality may influence the covariation between early-life growth rate and hunting mortality. Comparing early-life growth rates in an experimental population where the hunting pressure is manipulated could provide further insight into whether hunting indeed increases early-life growth rates, offering promising avenues for research.

## Electronic supplementary material

Below is the link to the electronic supplementary material.

Supplementary file1 (DOCX 55 kb)

## Data Availability

The data used in our analysis will be made available upon publication of this study.
