# Supplementary material for: Grow fast at no cost: no evidence for a mortality cost for fast early-life growth in a hunted wild boar population
Source: Oecologia. 2020 Apr 2;192(4):999–1012. doi: 10.1007/s00442-020-04633-9 (PMC7165149; doi:10.1007/s00442-020-04633-9)
Supplement: Supplementary file 1 — Supplementary file1 (DOCX 55 kb) [file 442_2020_4633_MOESM1_ESM.docx]

Supplementary Materials for the article:

“Grow fast at no cost: No evidence for a mortality cost for fast early-life growth in a hunted wild boar population”

Lara Veylit, Bernt-Erik Sæther, Jean-Michel Gaillard, Eric Baubet, Marlène Gamelon

*****Correspondence author: [lara.veylit@ntnu.no](mailto:lara.veylit@ntnu.no)

**Appendix S1: Specific information from the literature linking early-life growth to survival**

Table S1

|  |  | Effect | |  | |
| --- | --- | --- | --- | --- | --- |
| Species | Reference | Males | Females | | Quote demonstrating trade-off |
| Bighorn sheep | Bonenfant et al. 2009 | (0) | NA | | “Growing large horns early in life is not related to any consistent survival costs. |
| Dall sheep | Loehr et al. 2007 | (-) | NA | | “We found a negative relationship between horn growth rate and longevity for natural mortality in areas with little or no hunting.” |
| Stone sheep | Douhard et al. 2016 | (-) | NA | | “A decline in harvest age with rapid early horn growth was more marked in the high than in the low hunting area.” |
| Alpine ibex | Toïgo et al. 2013 | (0)^1^ | NA | | “(...) early horn growth did not negatively influence survival over most of the lifetime, indicating that males do not invest in horn growth at survival costs at least until 12 years of age.” |
| Alpine ibex | Bergeron et al. 2008 | (0) | NA | | “Our results suggest that males with fast-growing horns early in life were able to grow longer horns without any apparent longevity cost.” |
| Chamois | Bleu et al. 2014 | NA | (-) | | “(…) the best-fitting model included a negative effect of early horn growth on yearly survival of 8-12 years old females.” |
| Chamois | Corlatti et al. 2017 | (0) | (0) | | “Within the protected population, under pressure of sexual/natural selection, we found statistically non-significant negative trade-offs between early horn growth and survival in both males and females.” |
| Chamois | Corlatti et al. 2017 | (-) | (+/-)^2^ | | “(…) selection on early horn growth of culled individuals showed diametrically opposed sex-biased patterns, depending on the culling regime and hunters’ preferences.” |
| European mouflon | Kavčić et al. 2019 | (-) | NA | | “The mean age at death of rams with rapid early horn growth (> 50 cm) was 3.86 years, while it was 4.64 years for rams with slower early horn growth (< 50 cm).” |
| Three-spined stickleback | Lee et al. 2012 | (-) | (-) | | “These results demonstrate the growth–lifespan trade-off.” |
| Subantarctic fur seals | Chambellant et al. 2003 | (+) | (0) | | “(…) the GR_60_ value for male pups which survived to weaning is higher than for pups that died...The relationship between GR_60_ and SR_L_ was found to be not significant in female pups.” |
| Subantarctic fur seals | Beauplet et al. 2005 | (+) | (+) | | “We detected a significant positive relationship between pup preweaning growth rate and individual post-weaning survival and interestingly, this effect was greater for males than for females." |
| Speckled wood butterfly | Gotthard et al. 1994 | (-) | (-) | | “(…) the results of the longevity study indicate that a shorter life span may represent a cost of high growth rates...” |
| Perch | Metcalfe and Monaghan 2003; Craig 1980 | (-) | NA | | “(…) the average early growth rate of a year-class is the strongest predictor of its subsequent adult mortality rate, with those year-classes exhibiting the fastest early growth having the shortest life expectancy.” |
| European plaice | Jorgensen and Holt 2013 | NA | (-) | | “Fish tend to take more risk to accelerate growth prior to maturation, despite increased mortality.” |
| Rhesus Macaques | Nuñez et al. 2015 | (+) | (+) | | “For the heaviest and fastest-growing juveniles, this can mean a five to ten-fold decrease in hazard.” |
| Wild type and Norway rats | Rollo 2002 | (-) | (-) | | “Peak body mass (which reflects juvenile growth rates) was negatively associated with longevity within both species.” |
| Tasmanian snow skinks | Olsson and Shine 2002 | (-) | (-) | | “When later released into the wild, the individuals that grew more rapidly as neonates experienced much higher mortality than did slower-growing conspecific" |

^1^Early-life growth was not related to survival until late life, when early horn growth incurred a survival cost.

^2^ The culling regime and hunter preference determined survival patterns in the two harvested populations

**Table S1**: Studies linking early-life growth rates to survival (see Table 1 in the text). Specific information from the text is included to identify the relationship between early-life growth rate and survival.

**Appendix S2: Growth rate calculation: testing the assumption of linearity of growth rates**

Individual early-life growth rates for 516 males and 475 females were included in the analysis. These rates were calculated using an alternative method (Method 2), which does not have as strict an assumption of linearity as Method 1 (outlined in the text). Instead of estimating growth rate from the first and the last body mass measurements (Method 1) and requiring 2 body mass measurements, we took into account all the successive body mass measurements such as:

$$\text{G}\text{i }\text{=}\frac{\sum_{\text{j=1}}^{\text{n}} \frac{\text{W}\text{(j+1) }\text{– }\text{W}\text{j}}{\text{T}\text{(j+1) }\text{– }\text{T}\text{j}}}{\text{C}}$$

where the sum of each recorded body mass (*W*) subtracted by the body mass measurement recorded at the previous capture event is then divided by the time that elapsed between the two captures in days (*T)* divided by the number of captures (*C*). This method thus uses the average of successive individual growth rates from the first capture to the last measure of the wild boar when their weight was less than 20 kg (what we have defined as the period of early-life growth; see Gaillard et al. 1992). These two methods for calculating individual growth rates (G_i_) of individuals captured at least 3 times were compared using a linear regression. The sample size for the two methods were therefore different (Method 1 N = 991, Method 2 N = 377) as Method 2 required one capture more than Method 1. The relationship between the methods provided a R^2^ of 0.79 indicating a very good match between growth rates estimated with Method 1 and Method 2. Method 1 was chosen for our analysis as it was comparable to Method 2 and allows for the inclusion of more individuals in the analysis (for Method 2, the number of males = 208 and females = 169). Using Method 2, for males, the average early-life growth rate was 64.69 g/day (minimum = -25.19 g/day, maximum = 131.41 g/day) and for females, it was 59.77 g/day (minimum = -11.96 g/day, maximum = 119.80).

**Fig. S2** Linear relationship between growth rates calculated using two methods. Method 1 assumes growth rate is linear in early life and can be applied when only two measures of body mass are available. Growth rates from Method 1 were used in the analyses. Method 2 is an average of growth rates for a given individual and requires at least three measurements and weakens the assumption that early-life growth rate is linear.

**Appendix S3: Matrices for overall and cause-specific mortality models**

*Overall mortality models*

Matrix showing transition probabilities from time *t* (rows) to time *t+1* (columns) for overall mortality models. Three states were considered (*A* or alive, *M* or overall mortality, and *D* or already dead at time *t+1*).

$$\begin{matrix} A & M & D \end{matrix}$$

$$\begin{matrix} A \\ M \\ D \end{matrix}\left[\begin{matrix} 1-M & M & 0 \\ 0 & 0 & 1 \\ 0 & 0 & 1 \end{matrix}\right]$$

Matrix showing recapture (*p*) and recovery (*r*) probabilities from time *t* (rows) to time *t+1* (columns) for overall mortality models. Three states were considered (*ND* or not detected, *A* or alive, and *M* or dead). Individuals not seen (0) could be recaptured (*p*) or recovered (*r*) at time *t+1* unless they were already dead (*D*).

$$\begin{matrix} ND & A & M \end{matrix}$$

*Cause-specific mortality models*

Transition matrix showing cause-specific mortality probabilities from time *t* (rows) to time *t+1* (columns). Transitions for the four given states (*A*, or alive, *Mh* for hunting mortality, *Mn* for non-hunting mortality, and *D* for already dead at time *t+1*) are given.

$$\begin{matrix} A & Mh & Mn & D \end{matrix}$$

$\begin{matrix} A \\ Mh \\ Mn \\ D \end{matrix}$ $\left[\begin{matrix} 1-Mh-Mn & Mh & Mn & 0 \\ 0 & 0 & 0 & 1 \\ 0 & 0 & 0 & 1 \\ 0 & 0 & 0 & 1 \end{matrix}\right]$

The events matrix for the probability of being recaptured (*p*) or recovered (*r*) at time *t+1* (columns) given the state at time *t* (rows).

$\begin{matrix} ND & A & Mh \end{matrix}$

$\begin{matrix} A \\ Mh \\ Mn \\ D \end{matrix}\left[\begin{matrix} 1-p & p & 0 \\ 1-r & 0 & r \\ 1 & 0 & 0 \\ 1 & 0 & 0 \end{matrix}\right]$

**Appendix S4: Growth rate classes for models including early-life growth rate as a categorical variable**

Table S4

| Age class | Males | Females | Sample size, males | Sample size, females |
| --- | --- | --- | --- | --- |
| 1 | -86.21-0 | -170.00-0 | 32 | 32 |
| 2 | 3.01-25.00 | 3.07-30.00 | 37 | 37 |
| 3 | 25.16-43.16 | 31.58-40.98 | 35 | 32 |
| 4 | 45.28-54.74 | 41.06-52.00 | 34 | 35 |
| 5 | 55.17-64.29 | 52.04-61.54 | 34 | 33 |
| 6 | 65.16-75.00 | 62.11-70.97 | 37 | 41 |
| 7 | 75.12-82.96 | 71.31-81.82 | 34 | 40 |
| 8 | 83.33-90.00 | 82.14-89.66 | 33 | 33 |
| 9 | 90.74-97.78 | 90.14-96.88 | 39 | 34 |
| 10 | 98.15-107.89 | 97.10-105.88 | 35 | 28 |
| 11 | 108.33- 116.67 | 106.06-113.46 | 33 | 32 |
| 12 | 117.50-127.55 | 114.07-125.00 | 35 | 30 |
| 13 | 128.07-136.96 | 125.68-134.02 | 35 | 30 |
| 14 | 137.14-153.58 | 135.16-143.86 | 35 | 21 |
| 15 | 154.41-214.29 | 146.30-226.19 | 28 | 26 |

**Table S4**: Minimum and maximum early-life growth rates (in g/day) for males and females from models that include growth rate as a categorical variable for age class one (juveniles), two (subadult), and three (adults). These classes were chosen to include approximately 32 individuals in each categorical early-life growth rate class. The resulting models depicted the relationship between either early growth and overall (Fig. 3) or cause-specific (Fig. 4) mortality.

**Appendix S5: Intercept and slope estimates for the selected models**

Table S5

| Age class | Intercept | Slope |
| --- | --- | --- |
| A |  |  |
| Males |  |  |
| M(1&2&3) | **0.93 (SE: 0.10)** | **-0.27 (SE: 0.10)** |
| Females |  |  |
| M(1) | **1.06 (SE: 0.12)** | **-0.11 (SE: 0.12)** |
| M(2&3) | **0.31 (SE: 0.15)** | **0.13 (SE: 0.15)** |
| B |  |  |
| Males |  |  |
| Mh(1&2&3) | **0.44 (SE: 0.11)** | **-0.17 (SE: 0.11)** |
| Mn(1&3) | -1.13 (SE: 0.37) | < 0.01 (SE: 0.32) |
| Mn(2) | **0.66 (SE: 0.25)** | **-0.60 (SE: 0.26)** |
| Females |  |  |
| Mh(1) | **0.89 (SE: 0.19)** | **0.05 (SE: 0.14)** |
| Mh(2&3) | **0.20 (SE: 0.21)** | **0.07 (SE: 0.19)** |
| Mn(1&2&3) | -1.59 (SE: 1.13) | -0.45 (SE: 0.35) |

**Table S5**: Overall (A) and cause-specific (B) mortality. Displayed are the intercept and slope estimates for the selected models (see Figs. 2 and 3) on the logit and generalized logit scales for overall and cause-specific mortality estimates, respectively. Age classes are denoted as one for juveniles (up to one year of age), two for subadults (one to two years old), and three for adults (more than two years old). Pooled age classes are indicated with “&” between them. *M* indicates overall mortality estimates, *Mh* indicates estimates for hunting mortality, *Mn* indicates non-hunting mortality estimates, and SE indicates the associated Standard Error. Bolded values do not include zero in the confidence interval.
